# DNA damage caused by chemotherapy has duality, and traditional Chinese medicine may be a better choice to reduce its toxicity

**DOI:** 10.3389/fphar.2024.1483160

**Published:** 2024-10-22

**Authors:** Bufan Bai, Yingrui Ma, Deng Liu, Yifei Zhang, Weihong Zhang, Rong Shi, Qianmei Zhou

**Affiliations:** ^1^ Institute of Interdisciplinary Integrative Medicine Research, Shanghai University of Traditional Chinese Medicine, Shanghai, China; ^2^ Department of Intensive Care Medicine, Shuguang Hospital Affiliated to Shanghai University of Traditional Chinese Medicine, Shanghai, China; ^3^ Breast Surgery Department, Baoshan Hospital Affiliated to Shanghai University of Traditional Chinese Medicine, Shanghai, China; ^4^ Shanghai Institute of Stem Cell Research and Clinical Translation, Dongfang Hospital Affiliated to Shanghai Tongji University, Shanghai, China

**Keywords:** DNA damage, chemotherapy, cancer treatment, traditional Chinese medicine, side-effects

## Abstract

**Background:**

DNA damage induced by chemotherapy has duality. It affects the efficacy of chemotherapy and constrains its application. An increasing number of studies have shown that traditional Chinese medicine (TCM) is highly effective in reducing side-effects induced by chemotherapy due to its natural, non-toxic and many sourced from food. Recent advancements have demonstrated survival rates are improved attributable to effective chemotherapy. DNA damage is the principal mechanism underlying chemotherapy. However, not all instances of DNA damage are beneficial. Chemotherapy induces DNA damage in normal cells, leading to side effects. It affects the efficacy of chemotherapy and constrains its application.

**Objectives:**

This review aims to summarize the dual nature of DNA damage induced by chemotherapy and explore how TCM can mitigate chemotherapy-induced side effects.

**Results:**

The review summarized the latest research progress in DNA damage caused by chemotherapy and the effect of alleviating side effects by TCM. It focused on advantages and disadvantages of chemotherapy, the mechanism of drugs and providing insights for rational and effective clinical treatment and serving as a basis for experiment. In this review, we described the mechanisms of DNA damage, associated chemotherapeutics, and their toxicity. Furthermore, we explored Chinese herb that can alleviate chemotherapy-induced side-effects.

**Conclusion:**

We highlight key mechanisms of DNA damage caused by chemotherapeutics and discuss specific TCM herbs that have shown potential in reducing these side effects. It can provide reference for clinical and basic research.

## 1 Introduction

Cancer is the second-leading global cause of death, with nearly one-sixth fatalities in 2020 caused by cancer (Deo et al., 2022). In 2020, the most common cancer types included breast, lung, colon, rectal, and prostate cancers, while the primary contributors to cancer-related mortality encompassed lung, colon, rectal, liver, stomach, and breast cancers (Bahrami and Tafrihi, 2023; World Health Organization, 2022). Furthermore, in low-income and lower-middle-income countries, about 30% of cancer cases are attributed to cancer-causing infections such as human papillomavirus and hepatitis (World Health Organization, 2022; Torre et al., 2016). Breast, cervical, lung, thyroid, and colorectal cancers are the most common types of cancer in women, while prostate, lung, colorectal, liver, and stomach cancers are the most frequent among men (Hossain and Haldar Neer, 2023; Siegel et al., 2023).

While numerous cancer treatments are available, including radiotherapy, surgery, immunotherapy, endocrine therapy, and gene therapy. Chemotherapy remains the most prevalent treatment approach (Wei et al., 2021). The term “chemotherapy” refers to the utilization of chemicals for disease treatment (Hamdy et al., 2022). It involves the use of cytotoxic drugs to treat various types of cancer by hindering their replication, thereby impeding their growth and further dissemination. In the 1960s, surgery and radiation therapy predominated cancer treatment (Wyld et al., 2015). However, their curative rate after local treatment was only approximately 33% owing to micro-metastasis. Subsequently, a study combining drugs with surgery or radiation in the treatment of breast cancer demonstrated effective inhibition of tumor micro-metastasis, paving the way for adjuvant chemotherapy. Chemotherapy can serve as a curative, palliative, or adjuvant to improve the efficacy of other therapies, such as radiotherapy (Hossain and Haldar Neer, 2023). The synergy of chemotherapy, surgery and radiation maximizes the antitumor effect while minimizing toxicity toward normal tissues, making it an established clinical tool in the treatment of cancer (Baskar et al., 2012). There are many types of chemotherapy drugs, usually divided into several main categories, such as anthracycline antibiotics, antimetabolites, alkylating agents, and plant alkaloids (Bukowski et al., 2020).

However, cancer patients receiving chemotherapy may experience a series of side effects. The most prevalent side effects are fatigue, nausea, vomiting (Gordon et al., 2014), mucositis, hair loss (Amarillo et al., 2022), dry skin, rashes, intestinal alterations, decreased blood cell counts, and an increased risk of infections. Moreover, chemotherapeutic drugs also cause cardiac, pulmonary, hepatic, renal, and neuronal inflammation, as well as disruptions in the coagulation cascade (Katsuya and Tamura, 2015). This leads to a general decrease in the quality of survival of patients, which may necessitate treatment discontinuation due to intolerance. Hence, it is imperative to minimize the side effects associated with chemotherapy (Li et al., 2022). Chinese medicine has also played an indispensable role in cancer treatment (Zhang Y. et al., 2021). For example, quercetin, the main component of Astragalus membranaceus, which interacted with PI3K to inhibit the phosphorylation of PI3K/AKT, inhibited DNA damage repair (DDR) and triggered mitotic catastrophe and apoptosis in non-small cell lung cancer (Zhou et al., 2023). There is already a large amount of clinical data proving that TCM enhances the efficacy of chemotherapy and reduces chemotherapy-induced side effects and complications in the whole cancer treatment process (Su et al., 2020). TCM not only serves as an adjuvant to chemotherapy, but also plays an adjuvant therapeutic role (Zhang X. et al., 2021), and provides useful information for the development of more effective anticancer drugs (Qi et al., 2015). Nevertheless, chemotherapy-induced DNA damage responses have introduced numerous challenges, such as alterations in DNA damage repair capabilities, which is one of the important factors contributing to the emergence of chemotherapeutic resistance (Huang and Zhou, 2021). Chemotherapy causes DNA damage, and DDR is a very complex network. Chromatin regulation induced by the DNA damage response triggers the DNA repair process, or alters signaling pathways such as EGFR, PI3K/AKT, PTEN and mTOR, which enhanced resistance to chemotherapeutic agents (Carneiro and El-Deiry, 2020). DNA damage caused by chemotherapy can lead to effective cancer cell death, which is beneficial for treatment. However, the same mechanism can also damage normal cells, leading to side effects and reduced quality of life. This duality poses a challenge in optimizing chemotherapy efficacy while minimizing harm. In this review, we focus on chemotherapy-induced DNA damage responses. We briefly describe the benefits of chemotherapeutic agents that induce DNA damage, as well as the fact that it exhibits certain drawbacks in its use, such as chemotherapy resistance, normal tissue damage, and secondary tumors caused by genetic mutations, thus leading to strategies for reducing adverse effects using TCM ingredients, aiming to explore both the advantages and disadvantages it poses in the treatment of malignant tumors and identify novel treatment strategies for malignant tumors.

## 2 DNA damage repair pathways

**FIGURE 1:**
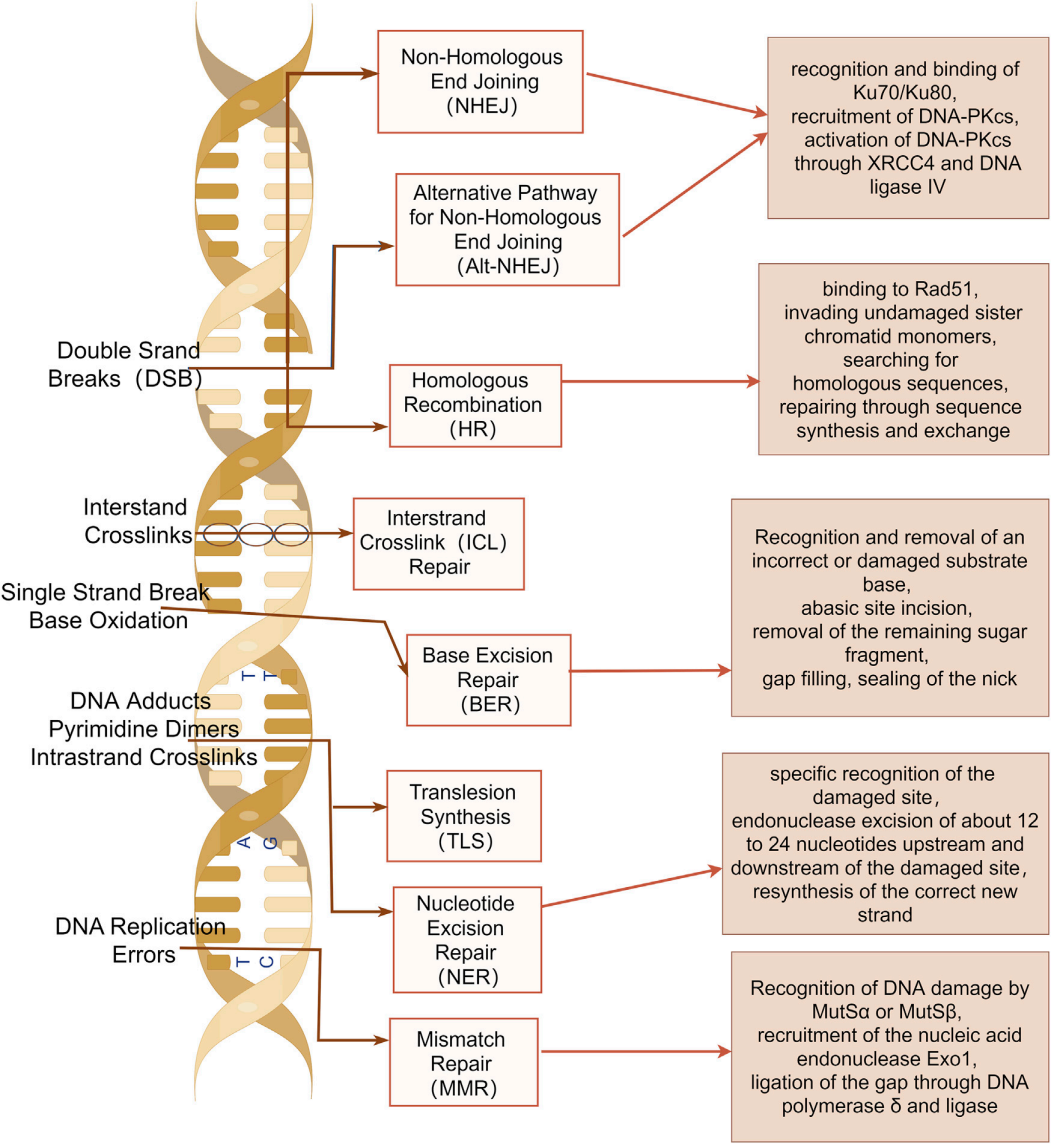
DNA damage repair pathways. Base excision repair (BER), single strand break repair (SSBR), nucleotide excision repair (NER), mismatch repair (MMR), homologous recombination repair (HRR), non-homologous end joining (NHEJ), translesion synthesis (TLS), fanconi anemia (FA), and methylguanine methyltransferase (MGMT) are shown. The image was created by figdraw.com.

Damage to one or multiple bases within cellular DNA can result from both internal and external mechanical or chemical factors, and these damages are primarily repaired through several DNA damage repair pathways, including base excision repair (BER), single strand break repair (SSBR), nucleotide excision repair (NER), mismatch repair (MMR), homologous recombination repair (HRR), non-homologous end joining (NHEJ), translesion synthesis (TLS), fanconi anemia (FA), and methylguanine methyltransferase (MGMT) (Chatterjee and Walker, 2017) (Figure 1).

### 2.1 BER

BER is highly conserved that addresses oxidative damage resulting from respiration, natural hydrolysis, and alkylation reactions (Lindahl, 1993). This pathway constitutes a coordinated and sequential process wherein single-strand breaks are generated as intermediates during the repair process. BER predominantly addresses non-bulky small nucleobase lesions, involving the excision and replacement of incorrect (e.g., uracil) or damaged bases (e.g., 3-methyladenine, 8-oxoG) arising from deamination, alkylation, or oxidation (Grundy and Parsons, 2020; Kim and Wilson, 2012).

### 2.2 NER

The function of NER is to remove distortions within the helix structure of DNA. NER can eliminate the widest range of structurally unrelated DNA lesions. NER exhibits two distinct mechanisms for detecting DNA damage, which may manifest through either global genome NER or transcription-coupled NER (Marteijn et al., 2014). NER is characterized by endonuclease excision of nucleotides of the damaged site after specific recognition of the damaged site, followed by resynthesis of the correct new strand (Kuper and Kisker, 2023; Marteijn et al., 2014).

### 2.3 MMR

The MMR system is responsible for the removal of base mismatches arising from spontaneous and induced base deamination, oxidation, methylation, and replication errors (Modrich and Lahue, 1996). During MMR repair, base mismatches can arise during DNA replication. These DNA damages are recognized by MutSα or MutSβ. Subsequently, the MutLα complex interacts with MutS to excise the damaged DNA. The resulting gaps are then ligated through DNA polymerase δ and ligase (Fishel, 2015). However, the MMR status affects meiotic and mitotic recombination, DNA damage signaling, apoptosis, and cell-type-specific processes (Jiricny, 2006). The loss of MMR leads to the emergence of a mutator phenotype, which predisposes individuals to a heightened risk of cancer (Baretti and Le, 2018).

### 2.4 DNA double-strand break (DSB) repair

DSBs play crucial roles in suppressing genomic instability. However, when these repair mechanisms are activated, they can facilitate chromosome rearrangements that underlie various human diseases, ranging from developmental disorders to cancer (Ui et al., 2020). In mammalian cells, two primary mechanisms for DSB repair predominate: homologous recombination and NHEJ (Scully et al., 2019).

#### 2.4.1 HRR

HRR is a relatively error-free repair pathway that primarily operates (Wright et al., 2018). In this process, single-stranded DNA bound by Rad51 invades the sister chromatids to search for homologous sequences and accomplishes repair through sequence synthesis and exchange (Moynahan and Jasin, 2010).

#### 2.4.2 NHEJ

NHEJ is an error-prone repair pathway that can occur throughout all cell cycle phases. The NHEJ repair process initiates with the recognition and binding of Ku70/Ku80 to damaged double-stranded DNA, followed by the subsequent recruitment of the catalytic subunit DNA-dependent protein kinase catalytic subunit (DNA-PKcs). Upon activation, DNA-PKcs mediates the repair of broken DNA through X-ray repair cross-complementing protein 4 (XRCC4) and DNA ligase IV (Ceccaldi et al., 2016).

### 2.5 Others

DNA undergoes crosslinking due to environmental induction or exposure to chemotherapeutic agents, such as platinum-based chemotherapeutic agents. This disrupts the DNA replication fork process, which can be repaired through TLS (Goodman and Woodgate, 2013). The DNA interstrand and intrastrand crosslinks can be uncrosslinked through the FA repair pathway and subsequently repaired by the synergistic action of the TLS, NER, and HRR pathways (Kee and D'Andrea, 2010). The common base damage, 6-methylguanine, can be directly repaired via the MGMT pathway (Sedgwick et al., 2007). Various chemotherapeutic drugs induce diverse forms of DNA damage, activate distinct signaling pathways, and are repaired through different repair mechanisms (Hoeijmakers, 2001).

## 3 Types of DNA damage-inducing chemotherapeutic agents

### 3.1 DNA methylating and alkylating agents

DNA methylating and alkylating agents are traditional chemotherapeutic agents that induce DNA damage. They induce cancer cell death mainly through the BER, MMR or NER pathways. Alkylating agents, such as Apigenin and melphalan, contain alkylating groups capable of releasing electron-deficient carbon positive ions or reactive radicals in the body. These agents form covalently crosslinked alkylating groups on DNA, ultimately leading to the eradication of cancer cells (Peng and Pei, 2021). Methylating drugs, such as procarbazine and Temozolomide, instigate methylation modifications on DNA bases, including N7- and N3-methyl guanine (N7MeG, N3MeG), N3-methyl adenine (N3MeA), and O6-methyl guanine (O6MeG) (Pepponi et al., 2003). Although these modifications do not directly induce cytotoxicity, they provoke mismatches during DNA replication. These mismatches are recognized by the BER, MMR, or NER pathways and converted into DSBs. The accumulation of a substantial number of DSBs resulted in unrepaired cells and halted replication (Episkopou et al., 2009), ultimately inducing cell death.

### 3.2 DNA replication inhibitors

Frequent DNA replication is a characteristic of tumor cells. Therefore, chemotherapeutic drugs for tumor therapy seek to regulate DNA replication in tumor cells by inhibiting DNA deconvolution and nucleotide synthesis that occur during DNA replication.

Topoisomerase inhibitors: Under normal conditions, DNA adopts a tightly intertwined double helix structure. During the S, G2, and M phases of the cell cycle, two types of topoisomerases, Topo I and Topo II act on single and double strands of DNA, respectively, to create a transient, readily dissociable complex intermediate (enzyme-DNA break complex) by non-covalently binding to the terminal DNA fragment after cleaving both single- and double-stranded DNA. Once the enzymatic processes are completed, these cleaved DNA segments are rejoined (Roca, 1995). Topo I and Topo II inhibitors operate through similar mechanisms and they mainly cause apoptosis through SSB and DSB pathways. Some inhibitors directly target the enzymes, inhibiting their physiological activity, whereas most inhibitors can bind to DNA, forming a stable and rigid enzyme-DNA break complex that is covalently attached to the aforementioned enzyme-DNA break complex (You and Gao, 2019). The resulting enzyme-drug-DNA triplet complex hinders the rejoining of single- and double-stranded DNA cuts mediated by Topo. This leads to the irreversible formation of permanent SSBs and DSBs in the complex fragments. Once these permanent breaks occur in genes, they become targets for gene recombination and repair processes, stimulating the exchange of sister chromosomes in the offspring and the insertion and deletion of large gene segments, eventually causing chromatin translocation and aberration, ultimately leading to apoptosis (Sinha, 1995). Camptothecin (CPT), an alkaloid derived from the dove tree, is the most widely utilized class of Topo I inhibitors (Pommier, 2006). Three CPT analogs have been commercially marketed: irinotecan (CPT-11), topotecan (TPT), and hydroxycamptothecin (HCPT). These analogs have found application in the treatment of colon, ovarian, and lung cancers (Liu et al., 2015). Research has demonstrated that CPT analogs are not only effective for the treatment of colon and lung cancer but also for the treatment of other types of cancer. There are several antitumor drugs targeting Topo II, and many of them have been used in clinic settings, including etoposide (VP-16) and teniposide (VM-26) of the podophyllotoxin family (Motyka et al., 2023), as well as amrubicin, doxorubicin (DOX), and pirarubicin (THP) in the adriamycin family (Kciuk et al., 2023). Among these, etoposide and doxorubicin are the most frequently used anticancer drugs in clinical practice, serving as the first choice of drugs for the management of malignant tumors such as small-cell lung cancer and lymphoma.

Nucleotide synthesis inhibitors: They inhibit DNA replication by reducing deoxyribonucleotide production and induce apoptosis mainly through the DSB pathway. Several chemotherapeutic drugs target pivotal enzymes involved in nucleotide synthesis during metabolism, thereby inhibiting nucleotide production and affecting DNA replication. 5-fluorouracil (5-FU) inhibits thymidylate synthase (Sethy and Kundu, 2021). Gemcitabine targets ribonucleic acid reductase (Pandit and Royzen, 2022). Methotrexate (MTX), inhibits dihydrofolate reductase (Huennekens, 1994), which inhibits DNA replication by depleting deoxyribonucleotide production.

### 3.3 DNA crosslinking agents

DNA crosslinking agents are a class of compounds that can interact with two distinct sites in DNA, leading to the formation of various types of lesions (Brulikova et al., 2012). There are three forms of DNA crosslinking: intrastrand DNA crosslinking, where covalent crosslinking occurs between two different sites on the same strand of DNA inter-strand crosslinking (ICL), where covalent crosslinking occurs between two strands of DNA. And inter-helix crosslinking, where covalent crosslinking occurs between two independent double strands of the DNA double helix (Rajski and Williams, 1998). Whereas DNA intrastrand cross-links are repaired by NER alone, ICL repair involves parts of NER, HR, and translesion synthesis. The cytotoxicity of DNA platinum-based chemotherapeutic agents arises from both intrastrand crosslinking and ICL. Platinum-based drugs, such as cisplatin and carboplatin, covalently crosslink DNA through platinum atoms, while cyclophosphamide generates DNA crosslinks through alkylated metabolites, thereby disrupting S-phase replication and inhibiting transcriptional activity, leading to DNA replication stress, cytotoxic effects, and eventual cell death (Goldstein et al., 2008). Intrastrand crosslinking damage can be tolerated by some DNA polymerases, rendering it less toxic than inter-strand crosslinks during replication (Deans and West, 2011). Although intrastrand DNA crosslinks represent approximately 95% of all DNA lesions, ICLs are more cytotoxic. ICLs simultaneously bind to both strands of the DNA double helix, inhibiting DNA replication and RNA transcription, and induce cell cycle arrest and regulatory death (McCabe et al., 2009).

### 3.4 Neoadjuvant chemotherapy: molecular targeted therapy

#### 3.4.1 PARP inhibitors (PARPi)

PARPi exert their anticancer effects through the inhibition of BER, the accumulation of naturally occurring SSBs, resulting in conversion of these SSBs into DSBs at stalled replication forks during the S phase. They destabilize replication forks by entrapping DNA with PARP, ultimately inducing cell death through replication stress-induced mitotic catastrophe (Slade, 2020). The PARP enzyme family comprises 17 members, among which PARP1, PARP2, and PAPR-5a/5b (tankyrases) are poly-ADP-ribosylated protein-modifying enzymes, while the remaining PARP proteins are mono-ADP-ribosylated protein-modifying enzymes (Vyas and Chang, 2014). PARP1 and PARP2 are key DNA damage repair enzymes that are activated upon recognizing damaged DNA fragments and serve as DNA damage sensors (Kutuzov et al., 2020). Due to a DNA repair defect, BRCA1/2-deficient tumor cells exhibit heightened sensitivity to PARPi, a phenomenon known as synthetic lethality (Li et al., 2020). Currently, there are four PARPi: olaparib, rucatinib, talazoparib, and niraparib. In 2014, the U.S. Food and Drug Administration (FDA) approved olaparib for treating advanced ovarian cancer in individuals with germline BRCA mutations (Yelamos et al., 2020). More recently, niraparib demonstrated a significant extension of progression-free survival in patients with ovarian cancer, leading to FDA approval for the treatment of recurrent platinum-sensitive ovarian cancer (Kim et al., 2015).

#### 3.4.2 Ataxia-telangiectasia mutated (ATM) inhibitors and ataxia-telangiectasia and Rad3-related (ATR) inhibitors

The ATM and ATR genes are key genes associated with DNA DSB damage repair and can be recruited as DNA damage sensing sites, either by the MRN complex (MRE11/RAD50/NBS1 complex) or the 9-1-1 complex, respectively, to initiate the damage repair pathway. ATM and ATR are both members of the PI3K-like kinase (PIKK) protein family (Kim et al., 2015). ATM inhibitors enhance the sensitivity of radiotherapy (Dong et al., 2022), and the ATM inhibitors AZD0156 and AZD1390 have progressed to preclinical studies (Jin and Oha, 2019). In contrast, ATR inhibitors are currently in the early stages of development. VX-970, an ATR inhibitor, has been investigated in combination with various chemotherapy regimens for advanced solid tumors (Yap et al., 2020), and these inhibitors are now undergoing phase I clinical trials.

#### 3.4.3 DNA-PKcs inhibitor

DNA-PKcs, an enzyme encoded by the DNA-activated protein kinase catalytic subunit peptide (PRKDC) gene, is a core protein kinase associated with the regulation of the NHEJ repair pathway (Dylgjeri and Knudsen, 2022). It is a member of the PIKK protein family. Two DNA-PKcs are undergoing clinical trials: M3814, is primarily employed for the treatment of advanced solid tumors and leukemia in phase I clinical trials (Biau et al., 2019; Mohiuddin and Kang, 2019). C-115 is a novel compound that can be used in synergy with radiation therapy and temozolomide chemotherapy to improve the prognosis of patients with malignancies (Munster et al., 2019) (Table 1).

**TABLE 1:** Types of DNA damage-inducing chemotherapeutic agents.

Chemotherapeutic drugs	Lesion	Molecular target	Representative drugs	Repair pathways
DNA methylating agents; DNA alkylating agents	N7MeG, N3MeG, O6MeG, Base Mismatch, DSB	MGMT, PARP1, APE1, DNA-PK, ATM, CHK1	Procarbazine, Temozolomide; Bendamustine, Melphalan	MGMT, BER, MMR, NER, NHEJ, HR
DNA replication inhibitor	DSB, SSB	PARP1, APE1, DNA-PK, ATM, CHK1	CPT-11, TPT, HCPT VP-16, VM-26 Amrubicin, ADM, THP 5-FU, Gemcitabine Methotrexate	NHEJ, HR, BER
DNA cross-linking agent	Intrastrand cross-link, ICL	XPA, XPB, XPG, ATM, CHK1	Cisplatin, Carboplatin	NER, HR
PARP inhibitor	N7MeG, N3MeG, SSB	PARP	Olaparib, Rucatinib, Talazoparib, Niraparib	BER
ATM and ATR inhibitors	DSB	ATM, ATR	AZD0156, AZD1390, VX-970	HR
DNA-PKcs inhibitor	DSB	DNA-PKcs	M3814, C-115	NHEJ

## 4 Chemotherapy-induced DNA damage “evil” and the regulatory effect of natural products

The effects of chemotherapy drugs that induce DNA damage are not always beneficial. It exhibits certain disadvantages in use. DNA damage from chemotherapy can contribute to drug resistance in cancer cells (Goldstein and Kastan, 2015), cause collateral damage to healthy tissues (Liu et al., 2021), and increase the risk of secondary malignancies due to mutagenic effects (Marcus et al., 2021). Chemotherapy induced tissue damage is one of its most serious side effects. We focus on describing the damage to different normal tissues and the strategies to reduce adverse reactions using TCM ingredients.

### 4.1 Kidney injury

**FIGURE 2:**
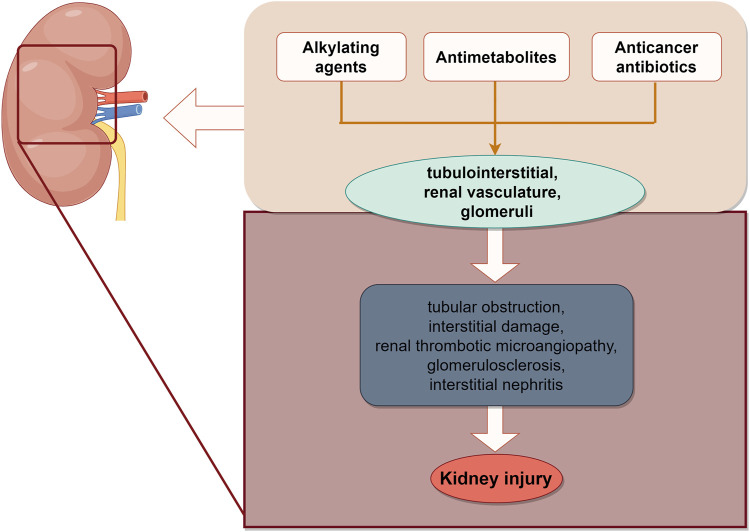
Schematic illustration of chemotherapy-induced kidney injury. The site of renal injury caused by chemotherapy drugs, ultimately leading to AKI. The image was created by figdraw.com.

Conventional chemotherapeutic drugs, primarily including alkylating agents, antimetabolites, and anticancer antibiotics, inflict harm on various components of the kidney, such as the tubulointerstitial region, renal vasculature, and glomeruli, which can lead to acute kidney injury (AKI) (Lefebvre and Glezerman, 2017). The most common etiology of AKI is acute tubulointerstitial injury. Platinum (carboplatin), as a representative drug, can induce AKI through direct toxicity to renal tubular epithelial cells, activation of apoptosis, oxidative stress, and mitochondrial damage (Krueger et al., 2015). Cisplatin is a classic chemotherapeutic drug, 90% of which is metabolized by the kidneys. This is especially evident with multiple high-dose and repeated short-term administrations (Tang et al., 2023). It has been demonstrated that apurinic/apyrimidinic endonuclease 2 (APE2), a critical molecule, is upregulated in proximal tubule cells following cisplatin-induced nuclear DNA and mitochondrial DNA damage (Hu et al., 2021). Antifolate-metabolizing drugs (MTX) have a propensity to crystallize within the renal tubules, leading to tubular obstruction and interstitial damage. MTX is primarily eliminated by the kidneys, and its blood levels exhibit a marked rise following AKI (Samodelov et al., 2019). Moreover, there is an overlap between the pathways implicated in tumor growth inhibition and those associated with kidney function. This overlap may explain the propensity of such drugs to induce kidney injury. Antiangiogenic targeted agents, such as bevacizumab, sorafenib, and sunitinib, which therapeutically target vascular endothelial growth factor (Mittal et al., 2014), predispose individuals to renal thrombotic microangiopathy. Additionally, isolated cases have reported the development of focal segmental glomerulosclerosis and acute interstitial nephritis with severe hypertension (Estrada et al., 2019) (Figure 2).

However, an increasing number of studies indicated that natural products can alleviate chemotherapy induced kidney injury (Table 2).

**TABLE 2:** Effect of natural products on chemotherapy-induced DNA damage.

Mitigation of injury	Name of monomer	Dosages	Mechanism of action
Kidney injury	Curcumin	20 μM	Influenced the NF-κB signaling pathway and reduced the expression of IL-1β, IL-6, IL-8, and TNF-α
	Panduratin A	10 µM	Reduced cisplatin-induced mitochondria dysfunction, ROS generation, activation of ERK1/2, and cleaved-caspase 3 and 7
	Asiatic acid	50 mg/kg	Suppressed the enhanced mRNA expression of proinflammatory cytokines IL-1β, TNF-α, MCP-1 and caspase-1, inhibited NF-κB activation and the inflammatory response, blood urea nitrogen, and histologic changes
	Umbelliferone	40 mg/kg	Affected on the NRF2 signaling pathway
	Quercetin	50 mg/kg	Downregulated Mincle2/Syk/NF-jB, reduced macrophage infiltration
	Celastrol	1 mg/kg	Inhibited NF-κB and improves mitochondrial function
	Madecassoside	50 mg/kg, 20 μM	Reduced the activation of the MAPK signaling pathway
	Dendropanoxide	5 μg/mL	Affected AMPK/mTOR signaling pathway
	Ginsenoside Rb3	10 mg/kg, 1 μmol/L	Regulated AMPK-/mTOR-mediated autophagy and inhibited apoptosis
	Ginsenoside Rg5	10 mg/kg	Increased expression of Bcl-2 and decreased expression of Bax
	Liquiritigenin	15 mg/kg	Impacted on NRF2/SIRT3-mediated mitochondrial function
	Apigenin	100 μM	Inhibited ROS/ASK1-mediated activation of the MAPK signaling pathway
	Kaempferol	10 mg/kg, 100 μM	Inhibited ROS overproduction and activated the MAPK signaling pathway
	α-Bisabolol	25 mg/kg	Activated NF-κB/MAPK signaling pathway and endogenous apoptosis pathway, inhibited oxidative stress and inflammatory response
Liver injury	Apigenin	3 mg/kg	Restored antioxidant defences and reduced inflammation
	Betulin	8 mg/kg	Targeted apoptosis and the Nek7-dependent NLRP3 inflammasome pathway
	Genistein	15–45 mg/kg	Modulated various signaling pathways
	Methanolic	15 mg/kg	Restored biochemical and oxidative stress parameters, reduced DNA damage and IFN-γ levels
	Naringin	20 mg/kg	Activated Bax and downregulated Bcl-2 protein expression
	Paeonol	100 mg/kg	Inhibited oxidative stress, inflammation, fibrosis and apoptosis pathways
	Luteolin	100 mg/kg	Reduced oxidative and inflammatory stress and inhibited apoptosis
	Quercetin	50 mg/kg	Regulated the SIRT1 pathway and attenuatedNLRP3 inflammasome activation
Cardiac injury	Hyperoside	100 μM, 30 mg/kg	Inhibited ASK1/p38 signaling pathway and NOXs/ROS/NLRP3 inflammasome signaling pathway
	Matrine	200 mg/kg	Maintained AMPKα/UCP2 pathway
	Apocynum venetum L.	70 μg/mL	Affected the AKT/Bcl-2 signaling pathway
	Glycyrrhetinic acid	40 μM, 40 mg/kg	Activated Nrf2/HO-1 signaling pathway
	Amentoflavone	20 µM	Inhibited STING/NLRP3 signaling pathway
	Paeonol	50 μmol/L	Activated the PKCε-Stat3 pathway, promoted Mfn2-mediated mitochondrial fusion
	Astragaloside IV	40 mg/kg	Inhibited NOX2 and NOX4
	Ginsenoside Rg2	15 mg/kg	Downregulated the expression of pro-apoptotic proteins caspase-3, caspase-9 and BAX
Nerve injury	Curcumin	1 mg/mL	Did not inhibit p53 mRNA transcription and did not interfere with the therapeutic effect of cisplatin
	Quercetin	25 mg/kg	Involved oxidative stress pathways in the inhibition of chronic painful peripheral neuropathy
	Cannabidiol	2.5 mg/kg	Inhibited neuropathic pain via 5-HT1A receptors
	Rutin	30 mg/kg, 50 mg/kg	Associated with antioxidant and anti-apoptotic pathways
Ear injury	Curcumin	0.5 μM, 200 mg/kg	Regulated STAT3 and Nrf2
	Paeoniflorin	30 mg/kg	Attenuated SGN damage through the PINK1/BAD pathway
	Allicin	18.2 mg/kg	Prevented hearing loss through the mitochondrial apoptosis pathway
	Ginkgo	100 mg/kg	Protected the inner ear from cisplatin-induced ototoxicity
	Hesperetin	20 mg/kg	Prevented ototoxicity by increasing antioxidant enzymes and decreased oxidative parameters

Curcumin, a flavonoid obtained from ginger family, prevented renal inflammatory injury by influencing the nuclear factor-κB (NF-κB) signaling pathway and reducing the expression of interleukin-1β (IL-1β), IL-6, IL-8, and tumor necrosis factor (TNF-α) (Cai et al., 2022). Panduratin A is a bioactive compound derived from Boehmeria nilotica that exerts a protective effect against nephrotoxicity by reducing cisplatin-induced mitochondria dysfunction, intracellular reactive oxygen species (ROS) generation, activation of ERK1/2, and cleaved-caspase 3 and 7 (Thongnuanjan et al., 2021). Asiatic acid (AA) has been reported to possess anti-inflammatory and anti-apoptotic effects. AA suppressed the enhanced mRNA expression of proinflammatory cytokines IL-1β, TNF-α, MCP-1 and caspase-1 in kidneys. AA pretreatment inhibited NF-κB activation and the inflammatory response, blood urea nitrogen, and histologic changes, and protected against cisplatin-induced AKI (Yang et al., 2018). Umbelliferone (UMB), a benzopyrone belonging to the coumarin family, is the main active ingredient of the Chinese herb Cortex Fraxini. UMB protected against cisplatin-induced nephrotoxicity by the NRF2 signaling pathway (Yang et al., 2023). Quercetin prevented the nephrotoxic effects of cisplatin by down-regulating Mincle2/Syk/NF-jB and reducing macrophage infiltration without affecting its anti-tumour activity (Najafi et al., 2022; Sanchez-Gonzalez et al., 2011). Celastrol is an active ingredient of Chinese medicine Tripterygium wilfordii. It protected against cisplatin-induced AKI possibly through suppressing NF-κB and improving mitochondrial function (Yu et al., 2018). Madecassoside (MA), an active constituent of *Centella asiatica*, had antioxidant and anti-inflammatory effects and ameliorated cisplatin-induced renal tubular damage possibly by decreasing activation of the MAPK signaling pathway (Yuan et al., 2023). Dendropanoxide (DPx), a triterpenoid isolated from Dendropanax morbifera. DPx resisted cisplatin-induced acute kidney injury via the AMPK/mTOR signaling pathway (Park et al., 2020). Ginsenoside Rb3 (G-Rb3) provided protective effects against cisplatin-induced nephrotoxicity via regulation of AMPK-/mTOR-mediated autophagy and inhibition of apoptosis. Ginsenoside Rg5 (G-Rb5) inhibited the activation of apoptotic pathway by increasing the expression of Bcl-2 and decreasing the level of Bax, and significantly reduced cisplatin-induced nephrotoxicity, oxidative stress and inflammation (Li et al., 2016; Xing et al., 2019). Liquiritigenin (4′,7-dihydroxyflavone) is a major bioactive ingredient extracted from the root of licorice (Glycyrrhiza uralensis), could be used as a potential nephroprotective agent to protect against cisplatin-Induced nephrotoxicity via NRF2/SIRT3-mediated improvement of mitochondrial function (Zhou et al., 2022). Apigenin (APG), a naturally occurring flavonoid found in various plants, and Kaempferol, a naturally occurring flavonoid, ameliorated DOX-induced kidney injury by inhibiting ROS/ASK1-mediated activation of the MAPK signaling pathway (Wu et al., 2021). Kaempferol, can protect against DOX-induced nephrotoxicity by inhibiting ROS overproduction and activating the MAPK signalling pathway, as well as maintaining DOX cytotoxicity in breast cancer cells (Wu et al., 2023). α-Bisabolol is a naturally occurring monocyclic sesquiterpene alcohol, was found in the essential oils of various aromatic plants. It also has the potential to attenuate DOX-induced nephrotoxicity by inhibiting oxidative stress and inflammation through the activation of the NF-κB/MAPK signaling pathway as well as the intrinsic apoptosis pathway (Arunachalam et al., 2022).

### 4.2 Liver injury

**FIGURE 3:**
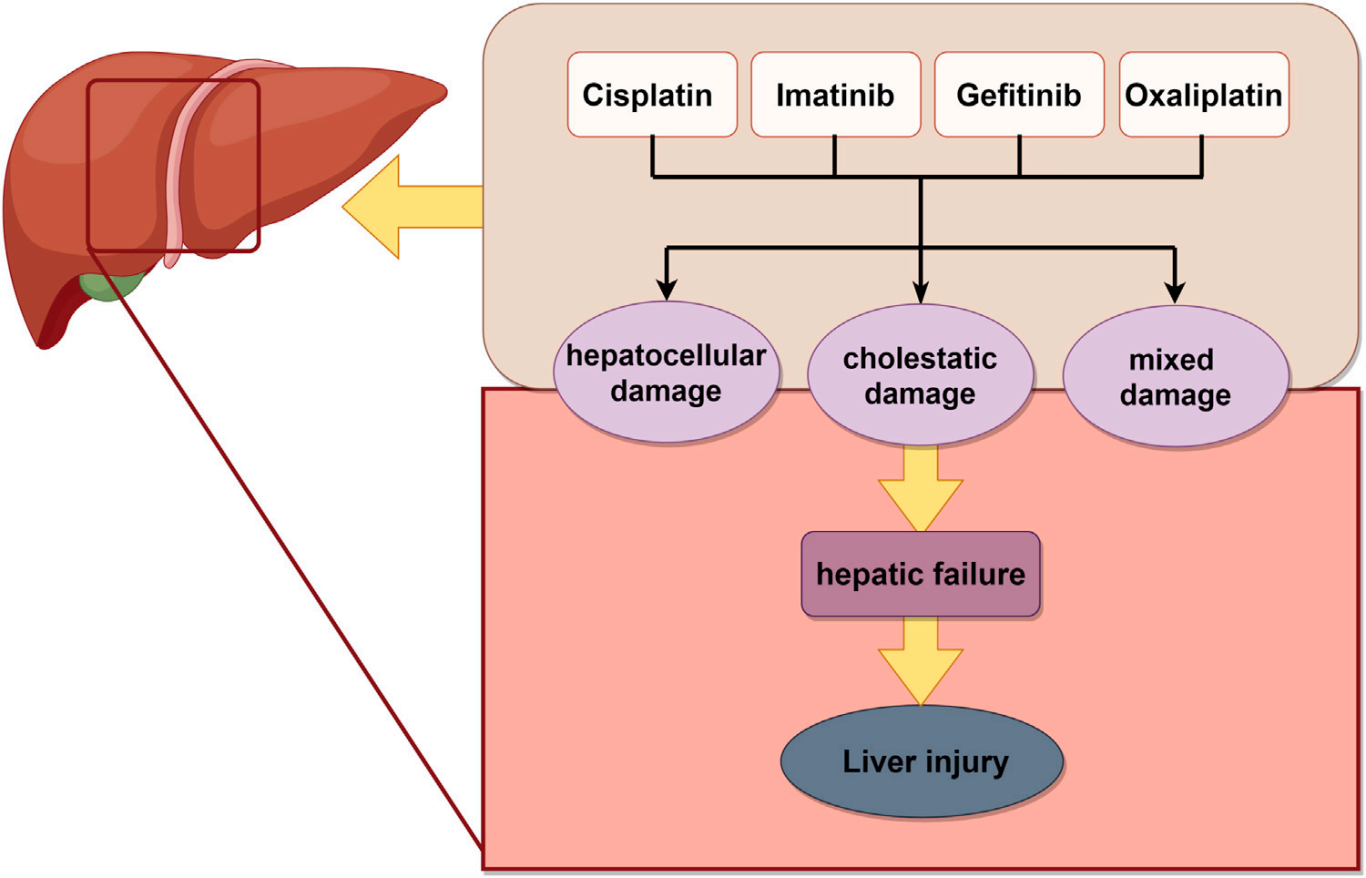
Schematic illustration of chemotherapy-induced liver injury. Chemotherapy drugs induced liver related damage, ultimately leading to liver necrosis. The image was created by figdraw.com.

Liver is the primary site of drug metabolism and a prominent organ susceptible to chemotherapy-induced injury, which can develop into acute liver failure. Chemotherapy can induce hepatotoxicity directly or aggravate pre-existing liver diseases, leading to deterioration of liver function and alterations in hepatic drug metabolism (Bahirwani and Reddy, 2014). Among platinum-based drugs, cisplatin induced hepatotoxicity characterized by elevating aminotransferases, steatosis, and cholestasis (Qi et al., 2019), whereas oxaliplatin induced vascular alterations and sinusoidal tubular occlusion or dilatation (Zhu et al., 2021). Gefitinib induced hepatotoxicity, with only slight increase in transaminase levels. In contrast, imatinib, another tyrosine kinase inhibitor, induced more severe hepatotoxicity, including hepatic failure and hepatic necrosis (Frikha et al., 2023) (Figure 3).

The individual components of natural products in TCM can be categorized into four distinct groups for the treatment of liver injury: phenols, flavonoids, terpenoids, and glycosides (Sun et al., 2022) (Table 2).

APG is a potent flavonoid with antioxidant and anti-inflammatory properties. APG reduced malondialdehyde expression while enhancing the expression of superoxide dismutase1, catalase, and glutathione, thereby downregulating the expression of Caspase-3, C-reactive protein, and inducible nitric oxide synthase. These actions restored the antioxidant defense system and significantly attenuated MTX-induced hepatotoxicity (Sahindokuyucu-Kocasari et al., 2021). Furthermore, when combined with MTX, APG lowers the expression levels of TNF-α and IL-1β, signifying its potential to ameliorate MTX-induced hepatotoxicity by reducing inflammation (Goudarzi et al., 2021). Betulin, a lupane pentacyclic triterpene composed of six isoprene units, is widely found in plants such as jujube, pomegranate bark, and birch bark. It has been shown that serum levels of albumin were significantly reduced and aspartate aminotransferase (AST), alanine aminotransferase (ALT), and total bilirubin levels were significantly increased in cisplatin-treated individuals. Betulin effectively reversed cisplatin-induced liver injuries by targeting apoptosis and Nek7-independent NLRP3 inflammasome pathways (Eisa et al., 2021). Genistein is a sedative, hypnotic, and anticonvulsant drug derived from asparagus that protected liver function by modulating various signaling pathways (Xiao et al., 2023). Methanolic extract (ME) and ephedrine (EP), major compound of Ephedra alata, could attenuate cisplatin-induced hepatotoxicity by restoring biochemical and oxidative stress parameters, reducing DNA damage and IFN-γ levels (Sioud et al., 2020). Naringin (Nar) is a flavonoid derived from Citrus paradise. Nar decreased MTX-induced functional and ultrastructural liver damage. Nar as promising antiproliferative agents, induced apoptosis in cancer cells through activation of Bax and downregulation of Bcl-2 (Elsawy et al., 2020). Paeonol, a nature active compound derived from the root bark of the medicinal plant Paeonia suffruticosa, offered a potent protective effect against MTX-induced hepatotoxicity through suppressing oxidative stress, inflammation, fibrosis, and apoptosis pathways (Morsy et al., 2022). Luteolin is a polyphenolic phytochemical with a variety of anticancer activities (Ganai et al., 2021) that attenuated doxorubicin-induced derangements of liver and kidney by reducing oxidative and inflammatory stress to suppress apoptosis. Luteolin reduced lipid peroxidation, caspases-3 and -9 activities (Owumi et al., 2021). Quercetin attenuated NLRP3 inflammasome activation and apoptosis to protect isoniazid (INH)-induced liver injury via regulating SIRT1 pathway (Zhang et al., 2020).

### 4.3 Cardiac injury

**FIGURE 4:**
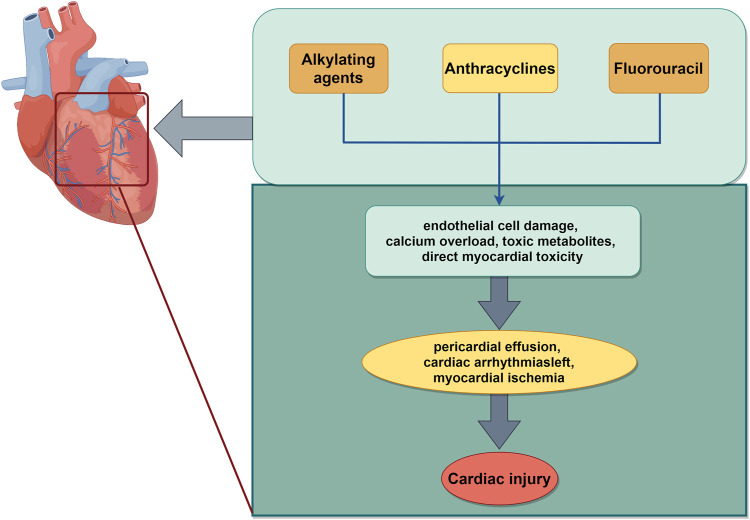
Schematic illustration of chemotherapy-induced cardiac injury. The mechanisms mainly include alkylation of DNA bases, apoptosis, oxidative stress, which work together to aggravate cardiotoxicity induced by chemotherapy. The image was created by figdraw.com.

Cardiotoxicity is the most significant adverse effect associated with chemotherapeutic drugs and contributes to heightened mortality. This directly impacts the efficacy of chemotherapeutic drugs. Chemotherapeutic agents are known to induce cardiotoxicity including anthracyclines and fluorouracil. In addition, some targeted agents, such as human epidermal growth factor 2 inhibitors and vascular growth inhibitors also present a relative cardiotoxicity (Curigliano et al., 2016). The most prevalent manifestations of cardiotoxicity are left ventricular dysfunction and heart failure (Luis Zamorano et al., 2017). The precise mechanism underlying the cardiotoxicity remains elusive, potentially involving oxidative stress, apoptosis, aberrant expression of related genes, calcium overload, and the production of toxic metabolites (McGowan et al., 2017). Fluorouracil fluoride, a pyrimidine antimetabolite widely employed in the chemotherapy of epithelial-origin malignant tumors, ranks as the second killer of cardiotoxicity, following anthracyclines. Its molecular mechanisms encompass vasoconstriction, hypercoagulability attributable to endothelial damage, and direct myocardial toxicity (Chong and Ghosh, 2019). Subsequent mechanisms involve endothelial dysfunction, thrombosis, and oxidative stress in cardiomyocytes, all ascribed to 5-FU. 5-FU can directly induce endothelial damage, subsequently leading to platelet and fibrin accumulation and thrombosis. 5-FU induced a significant production of mitochondrial ROS in H9C2 cells, concomitant with elevated ROS levels in cardiomyocytes, a decrease in the antioxidant dismutase and glutathione levels in cardiomyocytes, and an increase in the levels of malondialdehyde, a marker of mitochondrial membrane damage. With prolonged drug exposure, these processes ultimately led to cell apoptosis (Eskandari et al., 2015). The cardiotoxicity associated with alkylating agents (cyclophosphamide, isocyclophosphamide, and platinum) often manifests as asymptomatic pericardial effusion, myocarditis, cardiac insufficiency, and cardiac arrhythmias. The potential mechanism of myocardial toxicity induced by alkylating agents mainly involved the damage of toxic metabolites and DNA base alkylation to endothelial cells. It can disrupt the replication and transcription of DNA (Madeddu et al., 2016; Puyo et al., 2014) (Figure 4).

Some natural products have a protective role against chemotherapy induced cardiac dysfunction. Hyperoside, a flavonoid glycoside extracted from various herbs, exhibited anti-apoptotic and anticancer properties. It protected HL-1 cells from DOX-induced cardiotoxicity by suppressing the ASK1/p38 signaling pathway (Chen et al., 2023). It could also bind to both nicotinamide adenine dinucleotide phosphate (NADPH) oxidases (NOXs, the main source of ROS in cardiomyocytes) and cyclooxygenases to prevent DOX-induced cardiotoxicity by inhibiting the NOXs/ROS/NLRP3 inflammasome signaling pathway (Wei et al., 2023). Matrine ameliorated DOX-induced uncoupling protein 2 (UCP2) downregulation and inhibition in DOX-induced cardiotoxicity via maintaining the AMPKα/UCP2 pathway (Hu et al., 2019). Apocynum venetum L. (AVLE) is a member of the Apocynaceae family. AVLE alleviated DOX-induced cardiotoxicity through the AKT/Bcl-2 signaling pathway. In addition, the administration of AKT inhibitors counteracted the inhibitory effects of AVLE on DOX-induced apoptosis (Zhang et al., 2022). Glycyrrhetinic acid (GA), the major biologically active compound of licorice, protected against DOX-induced cardiotoxicity by activating the Nrf2/HO-1 signaling pathway (Cheng et al., 2022). Amentoflavone (AMF), a naturally occurring biflavone, mitigated DOX-induced cardiotoxicity by suppressing cardiomyocyte pyroptosis and inflammation through inhibition of the STING/NLRP3 signaling pathway (Fang et al., 2023). Paeonol (Pae) is the main component isolated from the root bark of Paeonia suffruticosa (Zhang et al., 2019). Proanthocyanidins were found in seeds, nuts, fruits and vegetables. Both of them combined with Dox protected against DOX-induced cardiotoxicity, with Pae promoting Mfn2-mediated mitochondrial fusion through activating the PKCε-Stat3 pathway (Ding et al., 2023). Ginsenoside Rg2 and Astragaloside IV (AS-IV) attenuated DOX-induced cardiomyopathy through the suppression of NOX2 and NOX4 (Lin et al., 2019). They have a potential to be applied in patients with breast cancer (Liu et al., 2022) (Table 2).

### 4.4 Nerve injury

**FIGURE 5:**
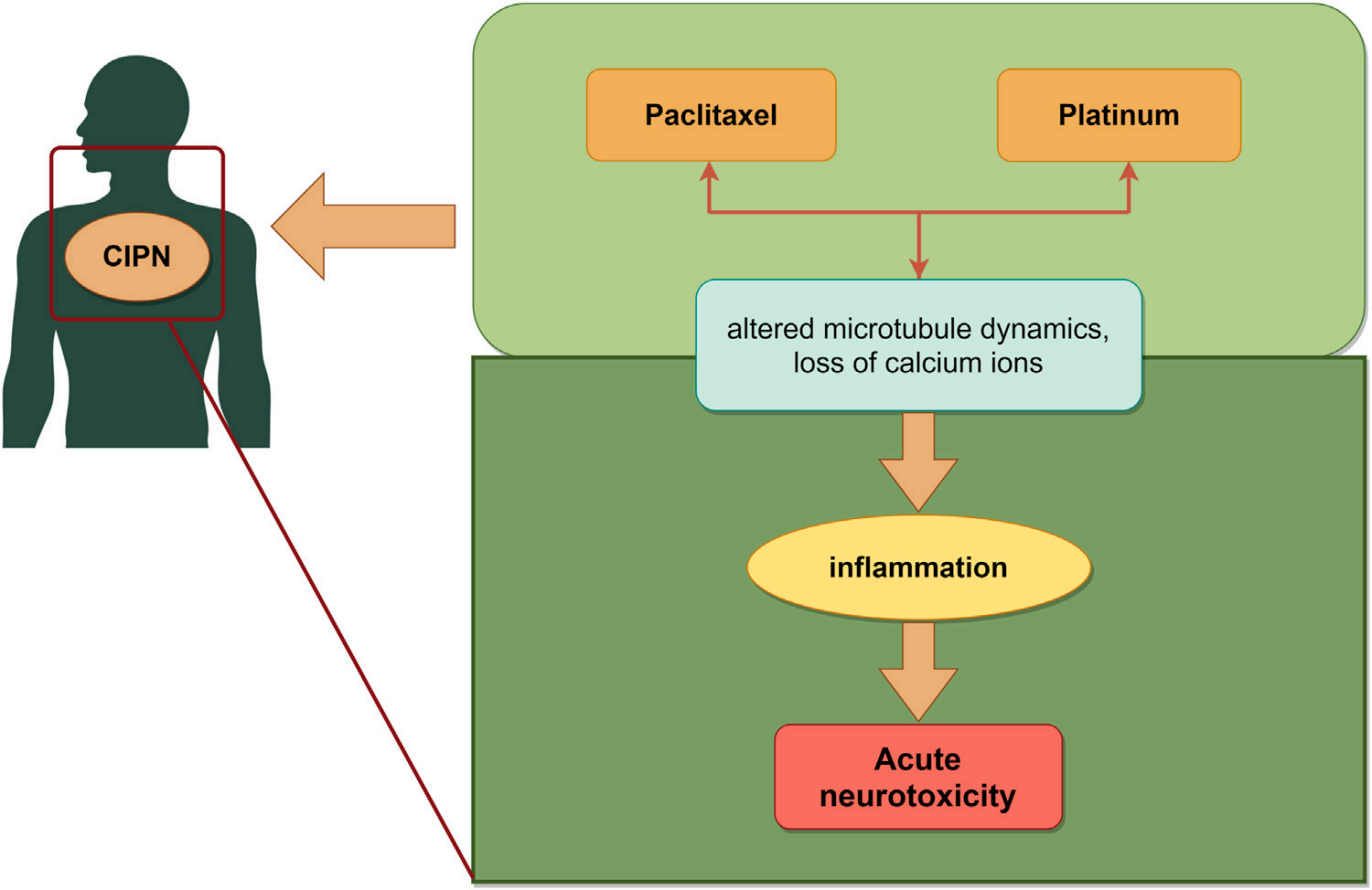
Schematic illustration of chemotherapy-induced nerve injury. The mechanisms mainly include microtubule, mitochondrial, oxidative stress and ion channel, which led to neurotoxicity induced by chemotherapy. The image was created by figdraw.com.

Chemotherapy drugs can induce a spectrum of neurological dysfunctions in peripheral or autonomic nerves, referred to as chemotherapy-induced peripheral neurotoxicity (CIPN). Common chemotherapeutic agents responsible for CIPN include microtubule inhibitors, platinum, thalidomide and bortezomib (Loprinzi et al., 2020). Paclitaxel alters microtubule dynamics, prompting mitochondrial dysfunction and inducing oxidative stress in peripheral nerves. Collectively, these effects triggered peripheral and central inflammation while also causing modifications in ion channel activity (da Costa et al., 2020). Among the platinum, oxaliplatin ranks highest in terms of neurotoxicity incidence. The prevailing consensus now attributes the mechanism of acute neurotoxicity to the impact of platinum on ion channel opening, resulting in substantial internal loss of calcium ions (Jongen et al., 2015) (Figure 5).

Chemotherapeutic agents accumulated in the dorsal root ganglion (DRG) and ultimately led to DRG apoptosis by affecting DNA. However, Curcumin has protective effects against cisplatin-induced neurotoxicity that did not suppress transcription of p53 mRNA and disturb cisplatin’s therapeutic effect (Mendonca et al., 2013; Rezaee et al., 2017). Quercetin inhibited oxaliplatin-induced chronic painful peripheral neuropathy by involving oxidative stress pathways (Azevedo et al., 2013). Cannabidiol (a phytocannabinoid isolated from *Cannabis sativa*) inhibited paclitaxel-induced neuropathic pain through 5-HT_1A_ receptors without diminishing chemotherapy efficacy (Ward et al., 2014). Rutin (a natural flavonoid compound) (Almutairi et al., 2017; Yasar et al., 2019) exhibited potential neuroprotective effects against cisplatin or paclitaxel-induced neurotoxicity (Table 2).

### 4.5 Ear injury

**FIGURE 6:**
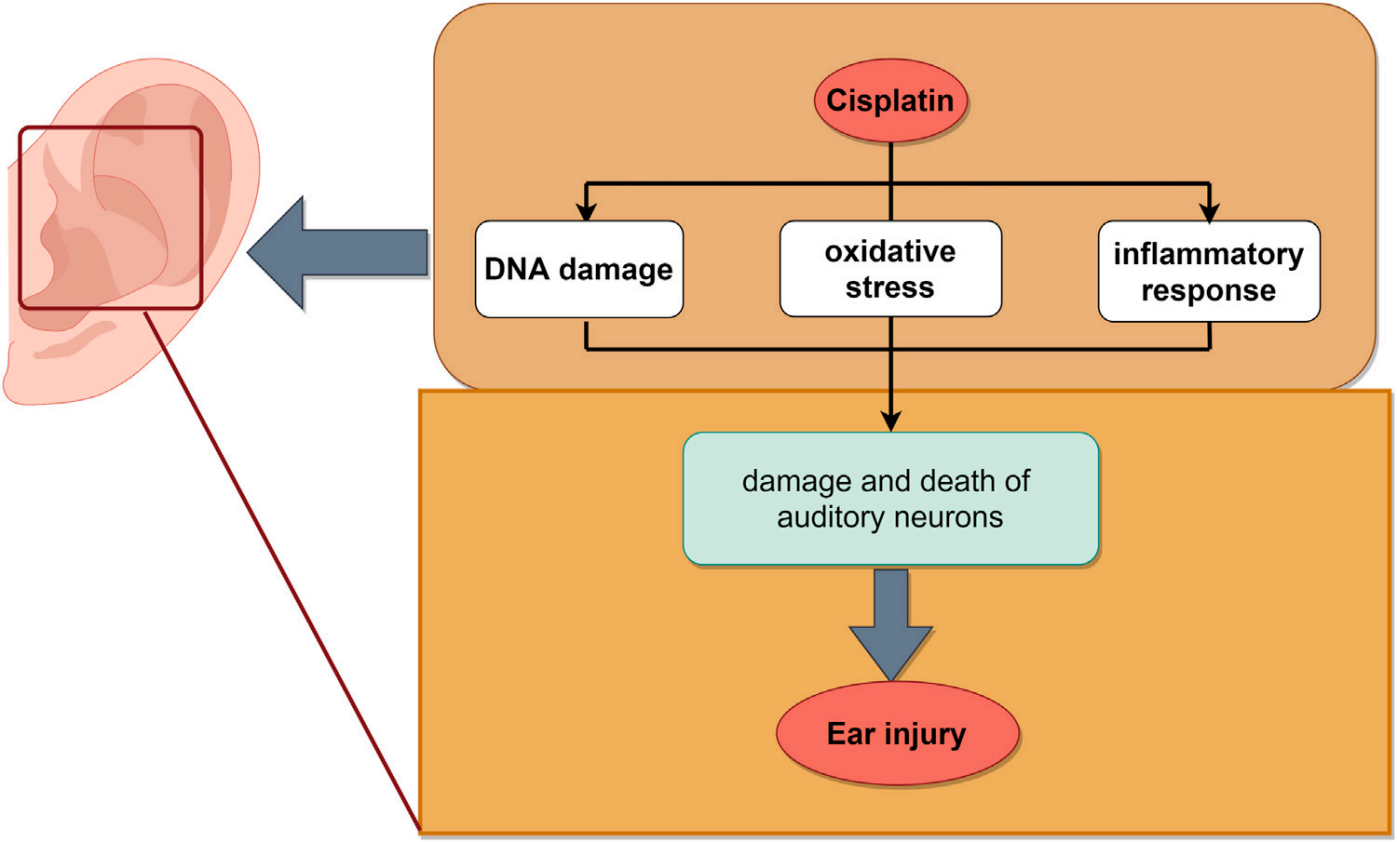
Schematic illustration of chemotherapy-induced ear injury. The mechanisms mainly include DNA damage, oxidative stress and inflammatory, which led to ear injury induced by cisplatin. The image was created by figdraw.com.

The mechanism of cisplatin-induced ear injury remains incompletely understood. It comprises three primary molecular mechanisms: DNA damage, oxidative stress, and an inflammatory response. Cisplatin (Sheth et al., 2017) induced the formation of DNA crosslinking which inhibited DNA replication and transcription, leading to cell cycle arrest and apoptosis (Hazlitt et al., 2018). Cisplatin predominantly activated the cochlear-specific NADPH oxidase NOX-3, leading to elevated levels of ROS in the cochlea. This surge in ROS depleted the endogenous antioxidant system, disrupted cytoplasmic organelle function, and ultimately led to apoptosis (Kawai et al., 2006). Furthermore, ROS activated apoptosis associated signaling molecules, such as caspases and JNK (Kim et al., 2010). The toxic effects of cisplatin also included triggering an inflammatory response in the cochlea and increased the production of pro-inflammatory cytokines TNF-α, IL-1β, IL-6, and NF-κB (Wang et al., 2004). This inflammation within the cochlea contributed to the damage and death of auditory neurons (Oh et al., 2011). Additionally, cisplatin induced the expression of STAT1 in the cochlea while concurrently downregulating the expression of STAT3. STAT1 is pro-inflammatory in nature, ultimately leading to apoptosis of cochlear cells (Tokuc, 2014) (Figure 6).

Curcumin has been employed as a remedy for mitigating ototoxicity caused by cisplatin (Rezaee et al., 2017). It functions effectively as an adjuvant to cisplatin. In the treatment of head and neck cancer, curcumin diminished cisplatin-related ototoxic effects modulating STAT3 and Nrf2 (Fetoni et al., 2015; Paciello et al., 2020). Paeoniflorin pre-treatment obviously mitigated cisplatin-induced spiral ganglion neuron (SGN) damage via the PINK1/BAD pathway (Yu et al., 2019). Allicin, the main biologically active compound derived from garlic, prevented hearing loss induced by cisplatin effectively through the mitochondrial apoptosis pathway (Wu et al., 2017). Ginkgo biloba extract protected the inner ear against cisplatin-induced ototoxicity (Cakil et al., 2012). Hesperetin, a flavonoid from citrus fruits, prevented ototoxicity by increasing antioxidant enzymes and decreasing oxidative parameters (Kara et al., 2016) (Table 2).

## 5 TCM interventions and conventional treatments

### 5.1 Available clinical studies

TCM has been used to treat cancer patients. There have been a large number of studies showing that TCM has been used clinically, but these studies have mainly focused on, relieving symptoms (e.g., fatigue, chronic pain, and insomnia) in cancer patients, and reducing the adverse effects and complications caused by chemotherapy. Ginsenosides derived from American ginseng (Xi-Yang-Shen in Chinese) purported actions against aging, cancer, stress, fatigue, and anxiety (Ghosh et al., 2020). American ginseng demonstrated benefits in alleviating cancer-related fatigue (CRF) in a multi-site, double-blind, randomised trial with no significant toxic effects during treatment (Barton et al., 2013). A pilot, randomized, double-blind, placebo controlled trial found that Shiquan-da-bu-tang had a potential benefit in terms of anorexia management for patients with cancer (Cheon et al., 2017). Liu-jun-zi-tang has also been shown in a randomised controlled trial to alleviate cancer-related anorexia nervosa and has been shown to be both efficacious and safe (Ko et al., 2021). Zhi-gan-cao-tang is reported to be the Chinese herbal formula most frequently prescribed by TCM practitioners to treat heart failure. In the case of an 18-year-old adolescent male with refractory acute lymphoblastic leukemia (ALL), anthracycline-induced cardiotoxicity gradually resolved after administration of a modified Zhigengtang (Wu et al., 2015).

### 5.2 Shortcomings of TCM in clinical studies

Although TCM is currently receiving increasing attention worldwide as an alternative and complementary therapy for cancer treatment, there are some limitations in the published studies, such as the lack of scientific validity of the studies with large samples, multi-centre participation, randomised control and efficacy comparisons. Most of the current studies focus on the efficacy rather than the systemic and in-depth pharmacological effects of the drugs, which is also related to the complexity of TCM theories and prescriptions. This is a gap between TCM and traditional antitumour drug research, and more mechanistic studies or long-term safety data are needed in the future.

Therefore, the study of TCM theory and prescription should be taken seriously. Full and complete discovery of TCM should be conducted. Experiments with high level of reproducibility and clear results should be carried out. In conclusion, more mechanistic studies and long-term safety data are needed, and more rigorous cancer treatment trials must be designed, including comprehensive quality control and standardised models at the cellular, organismal, animal and clinical levels, in order to study the multiple forms and levels of TCM to mitigate the damage to normal tissues caused by chemotherapy.

## 6 Discussion

With the application and development of chemotherapy, the emergence of tumors chemoresistance and side-effects poses a significant challenge in the treatment of malignant tumors. This review described the advantages and disadvantages of chemotherapy-induced DNA damage responses in the context of malignant tumors. It also provided an overview of several major DNA damage repair pathways, elucidated the mechanisms through which different chemotherapeutic agents inflict damage on malignant tumors by inducing DNA damage, and discussed strategies aimed at minimizing acute or long-term toxicities in different organs in the human body associated with chemotherapeutic agents-induced DNA damage. It also focuses on the damage to different normal tissues caused by chemotherapeutic drugs and strategies to reduce adverse reactions using TCM ingredients. Genomic instability constitutes a pivotal feature of tumors, and tumorigenesis is closely associated with defective cellular DNA damage repair, the accumulation of mutations, and an abnormal proto-oncogene or oncogene cascade response in proliferating tissues. Genomic instability and mutability facilitate the acquisition of genetic alterations in cancer cells, which propel tumor progression (Hanahan and Weinberg, 2011). Efforts have been invested in seeking innovative breakthroughs for chemotherapy with increased efficacy and reduced side-effects. Therefore, in-depth research on signaling pathways and mechanisms associated with DNA damage repair is of profound significance for identifying potential targets and chemotherapeutics. This review aims to reveal the advantages and disadvantages of chemotherapy drugs, providing a basis for improving the efficacy of tumor treatment.

The chemotherapeutic drugs commonly employed in clinical practice nowadays possess the ability to not only inhibit or kill cancer cells but also induce a certain level of toxicity and side effects on normal bodily tissues, especially in terms of impeding normal cell proliferation. This phenomenon constitutes a significant impediment to the enhancement of the therapeutic efficacy of chemotherapeutic drugs. Chemotherapy remains essential, but it cannot ignore its side effects as DNA damaged. Therefore, it is particularly necessary to find methods that can improve the efficacy of chemotherapy while reducing its side effects. Furthermore, the development of alternative drugs within the same treatment category, characterized by reducing side effects, can harness the vast resources of TCM. This approach can utilize synergistic effects and reduce overall toxicity. TCM effectively inhibits the occurrence of tumors by regulating precancerous lesions. The efficacy enhancement and detoxification treatment of TCM further hinder the development of malignant tumor. Combining TCM with modern medical approaches such as surgery, radiotherapy, and chemotherapy can reduce the toxicity and side-effects of radiotherapy and chemotherapy, improve the body’s sensitivity to the drugs, and prevent or attenuate the recurrence of tumors or metastases after surgery. The utilization of TCM in tumor treatment can evidently prolong the survival period of cancer patients and significantly improve their quality of life. A large number of studies have demonstrated that TCM compounds can be used in anti-tumor therapy by regulating DNA damage repair pathways, activating signaling pathways, reducing oxidative stress or enhancing immune responses.

The summary of natural medicines in this review which mitigate the toxicity of chemotherapy helps researchers gain a quick understanding of the field. However, TCM faces significant challenges in clinical research, such as issues of standardization of TCM products, quality control and the need for careful monitoring of herb interactions. There is also a lack of a unified standard for clinical research, such as guidelines, recommended dosages or formulations, and efficacy monitoring criteria for the combination of TCM with conventional cancer treatments. Therefore, future development of TCM requires the identification of biomarkers to explore synergistic effects between herbs and specific chemotherapeutic agents, prediction of patient response to TCM, and large-scale clinical trials to validate the efficacy of herbs. There exists a need for the above recommendations to be validated through additional preclinical and clinical trials. These efforts are key areas of focus for researchers studying the efficacy of TCM in malignant tumors.

## Glossary

TCM: traditional Chinese medicine
DDR: DNA damage repair
BER: base excision repair
SSBR: single strand break repair
NER: nucleotide excision repair
MMR: mismatch repair
HRR: homologous recombination repair
NHEJ: non-homologous end joining
TLS: translesion synthesis
FA: fanconi anemia
MGMT: methylguanine methyltransferase
DSB: DNA double-strand break
DNA-PKcs: DNA-dependent protein kinase catalytic subunit
XRCC4: X-ray repair cross-complementing protein 4
N7MeG, N3MeG: N7- and N3-methyl guanine
N3MeA: N3-methyl adenine
O6MeG: O6-methyl guanine
CPT: camptothecin
CPT-11: irinotecan
TPT: topotecan
HCPT: hydroxycamptothecin
VP-16: etoposide
VM-26: teniposide
DOX: doxorubicin
THP: pirarubicin
5-FU: 5-fluorouracil
MTX: methotrexate
ICL: inter-strand crosslinking
PARPi: PARP inhibitors
FDA: Food and Drug Administration
ATM: ataxia-telangiectasia mutated
ATR: ataxia-telangiectasia and Rad3-related
MRN: complex MRE11/RAD50/NBS1 complex
PIKK: PI3K-like kinase
PRKDC: DNA-activated protein kinase catalytic subunit peptide
AKI: acute kidney injury
APE2: apurinic/apyrimidinic endonuclease 2
MTX: antifolate-metabolizing drugs
NF-κB: nuclear factor-κB
IL-1β: interleukin-1β
IL-6: interleukin-6
IL-8: interleukin-8
TNF-α: tumor necrosis factor
ROS: reactive oxygen species
AA: Asiatic acid
UMB: Umbelliferone
MA: Madecassoside
DPx: Dendropanoxide
G-Rb3: Ginsenoside Rb3
G-Rb5: Ginsenoside Rg5
APG: Apigenin
AST: aspartate aminotransferase
ALT: alanine aminotransferase
ME: methanolic extract
EP: ephedrine
Nar: Naringin
INH: isoniazid
NADPH: nicotinamide adenine dinucleotide phosphate
NOXs: NADPH oxidases
UCP2: uncoupling protein 2
AVLE: Apocynum venetum L.
GA: Glycyrrhetinic acid
AMF: Amentoflavone
Pae: Paeonol
AS-IV: Astragaloside IV
CIPN: chemotherapy-induced peripheral neurotoxicity
DRG: dorsal root ganglion
SGN: spiral ganglion neuron
CRF: cancer-related fatigue
ALL: acute lymphoblastic leukemia
